# Consumption of Iron-Biofortified Beans Positively Affects Cognitive Performance in 18- to 27-Year-Old Rwandan Female College Students in an 18-Week Randomized Controlled Efficacy Trial

**DOI:** 10.3945/jn.117.255356

**Published:** 2017-09-27

**Authors:** Laura E Murray-Kolb, Michael J Wenger, Samuel P Scott, Stephanie E Rhoten, Mercy G Lung’aho, Jere D Haas

**Affiliations:** 1 Department of Nutritional Sciences, The Pennsylvania State University, University Park, PA;; 2 Department of Psychology and Cellular and Behavioral Neurobiology, University of Oklahoma, Norman, OK;; 3 Division of Nutritional Sciences, Cornell University, Ithaca, NY; and; 4 HarvestPlus, CIAT/Rwanda, Kigali, Rwanda

**Keywords:** biofortification, cognition, iron, Rwanda, women of reproductive age

## Abstract

**Background:** Evidence shows that iron deficiency in adulthood may affect cognitive performance, possibly by disrupting neurotransmitter regulation or brain energy metabolism. Women of reproductive age (WRA) are among those who are most vulnerable to iron deficiency; however, they have been largely ignored in the literature relating iron status to cognition.

**Objective:** Our aim was to determine the efficacy of iron-biofortified beans in improving cognition in WRA compared with control beans.

**Methods:** A double-blind, randomized intervention study was conducted in 150 women aged 18–27 y with low iron status (ferritin <20 μg/L). Women were randomly assigned to consume iron-biofortified beans (86.1 ppm iron) or control beans (50.1 ppm iron) daily for 18 wk. Iron status was assessed based on hemoglobin, ferritin, transferrin receptor, and body iron values and on cognitive performance on 5 computerized tasks at baseline and endline.

**Results:** Groups did not differ on any variables at baseline. Per protocol analyses revealed that consumption of the biofortified beans resulted in a 17% larger improvement in the speed of spatial selective attention; a nearly 7-fold larger improvement in the speed, a 68% greater improvement in the efficiency, and a >2-fold greater improvement in the specificity of memory retrieval; and a >2-fold larger improvement in the speed and a >3-fold larger improvement in the efficiency of memory search—all of which are relative to consumption of the control beans (*P* < 0.01 for all comparisons).

**Conclusions:** Cognitive performance is sensitive to iron status, and consumption of iron-biofortified beans for 18 wk improved cognitive performance, especially the efficiency of search and the speed of retrieval on memory tasks, in young adult women. This trial was registered at clinicaltrials.gov as NCT01594359.

## Introduction

Iron deficiency (ID) is the greatest single nutrient deficiency worldwide and most commonly affects infants, children, and women of reproductive age (WRA) (1, 2). Estimates from the WHO indicate that anemia affects ~43% of the world’s children <5 y of age, 29% of nonpregnant WRA, and 38% of pregnant women, although percentages vary widely by geographic region (3). In Central Africa, the prevalence of anemia is estimated at 48% for WRA and 56% during pregnancy (3). In Rwanda, 1 in 5 women between the ages of 15 and 49 y are anemic (4). Although anemia is not specific to ID, a recent report indicates that ~33% of anemia among WRA in sub-Saharan Africa is attributable to ID (5).

Included among the many consequences of ID are reduced capacity to perform physical work and negative changes in cognitive performance and affect (6). The literature describing animal studies reveals possible neural consequences of ID in 4 specific areas: myelination, morphology, metabolic activity, and monoamine biochemistry (7). With the possible exception of myelination [although see Daugherty et al. (8)], ID-related changes in neuronal morphology, metabolism, and monoamine synthesis and regulation are possible across the lifespan, suggesting that ID may exert effects on neural functioning well beyond infancy and childhood, including during the childbearing years. Nonetheless, although the prevalence of ID in WRA is significant to public health, research to date examining the relation between iron status and cognition has largely ignored this age group, focusing instead on infants and children.

Two observational studies designed to explore the relation between ID in the absence of anemia and executive functioning in WRA found that better iron status was associated with better planning ability (9, 10), attention (10), inhibitory control (10), and academic performance (11). Earlier studies exploring the relation between iron status and cognition in WRA reported decrements in performance on tests assessing learning, memory, and attention (12–16) and significant improvements with iron treatment (15). Yet this literature is still sparse: a meta-analysis examining the effects of iron supplementation on cognition in older children and adults recognized the lack of studies in this area and concluded that additional well-powered, blinded, randomized controlled trials are needed across all levels of iron status (17). Biofortification, the use of plant-breeding methods to enhance the micronutrient content of staple crops, is a promising intervention to alleviate the burden of ID in populations that may be difficult to reach with other interventions (18–22). Whether dietary intervention with an iron-biofortified staple food would be efficacious in improving cognitive function in adults is unknown.

Optimal cognitive functioning is an especially important factor in an educational setting where pressure to succeed is high. For a woman who is attending an institute of higher education, cognitive functioning affects all aspects of life and is an important factor in her ability to compete in the classroom and in the future workplace (23, 24). If ID is linked to cognitive abilities, and if WRA are highly vulnerable to ID, then they may be at a disadvantage when it comes to success in both academics and a career as a result of a treatable deficiency. This work tested the hypothesis that iron repletion by way of a biofortified staple food (beans) would produce improvements in cognitive performance in WRA.

## Methods

### 

#### Subjects.

**FIGURE 1:**
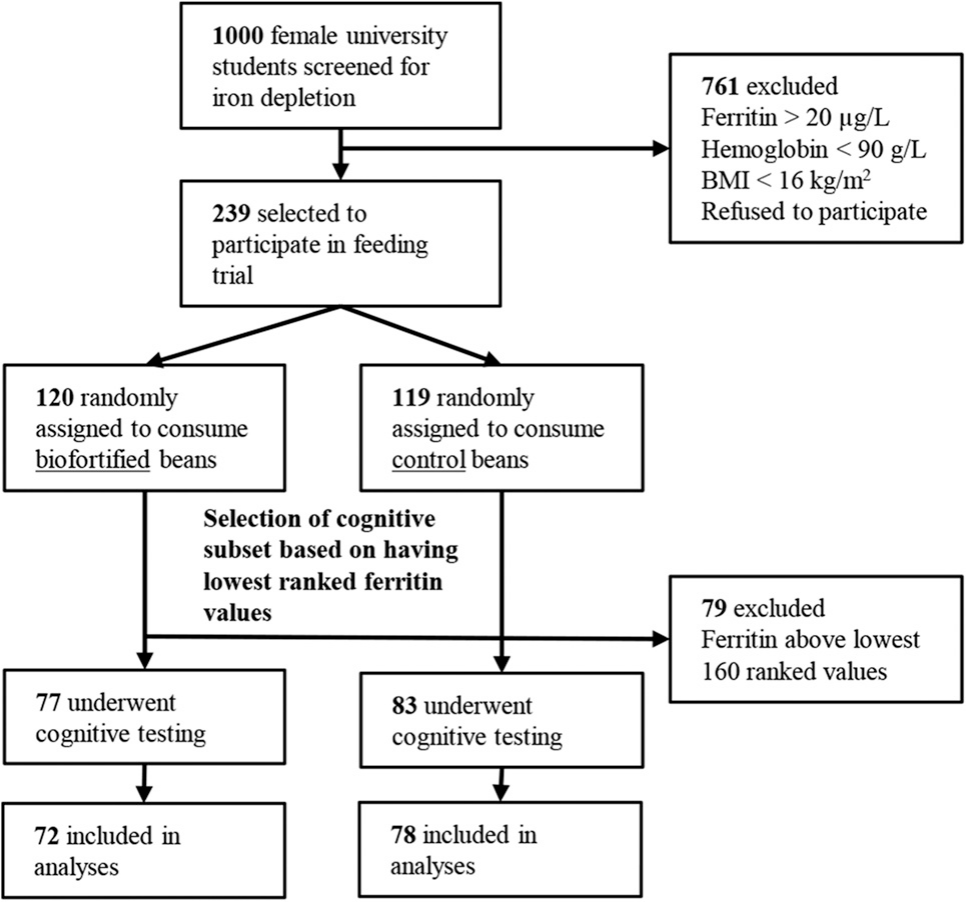
Diagram of the screening and selection processes for the randomized trial in Rwandan female college students.

Women attending the University of Rwanda, Huye campus, were recruited into the larger parent study (22) because they had depleted iron stores [serum ferritin (SF) <20 μg/L] but were otherwise healthy. Exclusion criteria were as follows: age <18 or >27 y, BMI (in kg/m^2^) <16, hemoglobin <90 g/L, SF >20 μg/L, any major medical conditions, use of iron supplements, use of medications that could interfere with dietary iron absorption, and pregnancy or lactation. Of 1000 women screened 2 mo before the start of the feeding trial, 239 were eligible and enrolled (22) (**Figure 1**). Of those enrolled, a subset of 160 subjects with the lowest baseline SF concentrations (assessed by ranking women independent of treatment assignment) was chosen to undergo cognitive testing, as this subgroup was thought to have the greatest potential to benefit from the intervention and, therefore, to demonstrate cognitive change. From the subset of 160 women with the lowest baseline SF concentrations, those with complete data (*n* = 150; 78 control, 72 biofortified) were included in the current analyses. Sample sizes were calculated based on the smallest effect size described by Murray-Kolb and Beard (15), assuming a 2-tailed 5% type I error rate and 90% power, and revealed that a sample size of 60 subjects/group would be sufficient to detect differences in cognitive outcomes.

#### Study design.

The study was a double-blind randomized efficacy trial. Beans were consumed from 7 January to 23 May 2013, for a total of 128 feeding days (just over 18 wk). Women were randomly assigned to 1 of 4 color groups. Two colors represented biofortified beans (86.1 ppm Fe) and 2 colors represented conventional beans (control; 50.1 ppm Fe) (Figure 1). Beans were consumed 2 times/d, at lunch and dinner, in a cafeteria setting. Based on pilot data to determine the acceptable portion size, 175 g beans (cooked weight) were given to each woman at each meal; women consumed beans ad libitum, with the option to return for an additional serving. Waste left on the plate was weighed after each meal to determine how much was consumed. Full methodological details of the beans and feeding trial are described elsewhere (22). Blood samples were obtained and cognitive tests were conducted at baseline and endline.

#### Laboratory analysis.

Whole-blood samples were collected from an antecubital vein by a trained phlebotomist at baseline and endline; hemoglobin in the samples was analyzed within 6 h of collection at Kanombe Military Hospital in Kigali. A second tube coated with heparin was collected for a complete blood count including hematocrit, mean corpuscular volume, mean corpuscular hemoglobin concentration, and red cell distribution width (Sysmex Automated Hematology Analyzer model XS-1000i). SF, serum transferrin receptor (TfR), C-reactive protein (CRP), and α-1-acid glycoprotein (AGP) were analyzed by the VitMin Laboratory in Willstaett, Germany, after a sandwich ELISA procedure (25). Total body iron (BI) was estimated as the ratio of TfR and SF, according to Cook’s equation (26). Laboratory samples were tested in batches by a senior technician, and instruments were calibrated daily based on standardized procedures. A constant 6 g/L was subtracted from hemoglobin values to account for altitude. Anemia was defined as hemoglobin <120 g/L; ID as SF <15 μg/L, TfR >8.3 mg/L, or BI <0 mg/kg; and inflammation as CRP >5.0 mg/L or AGP >1.0 mg/L.

#### Cognitive tests.

Each subject underwent a 60- to 90-min computerized cognitive testing session during the first 3 wk at baseline and the final 3 wk at endline. Trained research assistants gave test instructions in the local language (Kinyarwanda). Subjects were seated ~70 cm from the screen.

Five common measures of attentional and mnemonic functioning were used; these were selected on the basis of 2 criteria. First, we selected measures that tap the use of cortical systems and circuits that have been documented, in the literature describing either human or animal studies, to have some dependence on iron status. Second, we selected tasks that have been used extensively in the literature on human experiments in order to allow external validity to be checked.

**FIGURE 2:**
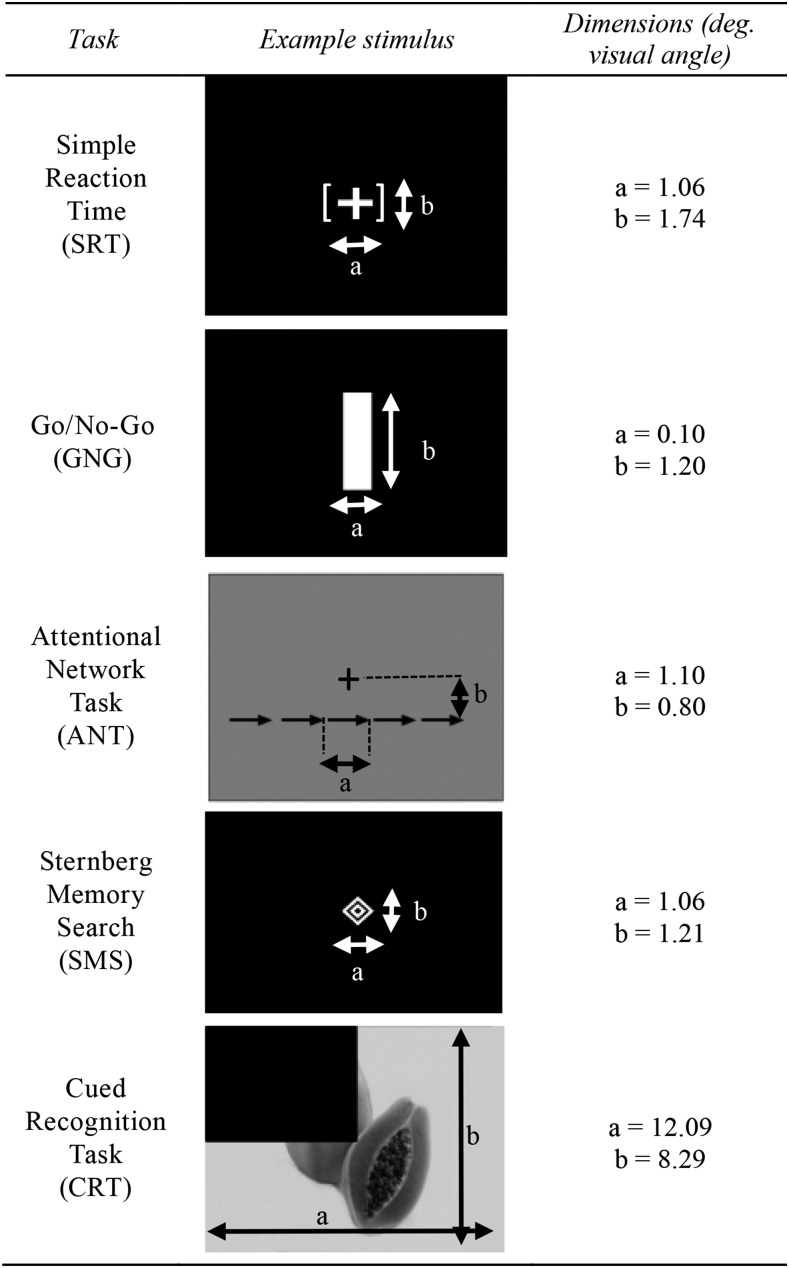
Example stimuli and dimensions for the 5 cognitive tasks administered to Rwandan female college students. deg., degree.

All of the tasks were computer-based, included a set of standardized explanatory instructions and practice trials, and were administered by local research assistants trained in common performance criteria. All of the tasks were developed and programmed by MJW using DMDX software (27); programs and stimuli are freely available upon request. The tasks were presented on Windows-based laptop computers with 36-cm (diagonal) displays, running at 2.5 GHz, with ≥4 GB of random access memory and ≥320 GB of hard disk storage. Stimulus onsets were synchronized to the vertical refresh rate of the monitor, and keyboard responses were timed to ±1 ms. Stimuli for all of the tasks were either grayscale images or white characters on a black background. Example stimuli for each of the tasks and the order in which the tasks were presented are shown in **Figure 2**. The dependent variables from each of the tasks are presented in **Supplemental Table 1**. Brief descriptions of the tasks are provided here; procedural details are presented in **Supplemental Table 2**.

The simple reaction time (SRT) task (28) provides an estimate of the speed of the simplest possible behavioral response to a visual stimulus; the task required the participant to press a button in response to the onset of the task stimulus (a simple string of characters). The task has limited attentional and mnemonic demands. The go/no-go (GNG) task (28) provides an estimate of the efficiency of sustained attention and the speed of simple attentional capture without the need to filter information from any immediately competing stimuli. The task required the participant to press a button in response to the presentation of an infrequent stimulus (either a horizontal or vertical bar, presented on 20% of the trials) and to withhold a response to a frequent stimulus (either a horizontal or vertical bar, presented on 80% of the trials). The attentional network task (ANT) (29) is a modified flanker task that provides an estimate of the effectiveness of 3 distinct components of attention. In each trial, the participant was presented with either an informative or an uninformative cue and was required to press a button to indicate whether an arrow in the center pointed to the left or right, while attempting to disregard flanking elements. The cued recognition task (CRT) is a modified version (30) of a classic visual recognition memory task (31) that estimates the speed, accuracy, and efficiency of recognition based on short-duration visual memory. Participants are presented with a set of pictures of common, easily named objects. After presentation of the memory set, an equal number of previously seen (old) and novel (new) items are presented, and participants indicate whether each item was in the original memory set or is new. The Sternberg memory search (SMS) task (32) estimates the speed and accuracy with which immediate visual memory can be searched. In each trial, participants were first shown a set of 1, 3, or 6 simple abstract (non-verbalizable) graphical symbols and were instructed to commit them to memory. Then a test stimulus was presented and the participant indicated whether they remembered the test stimulus from the preceding set of symbols.

#### Ethics.

All subjects provided written informed consent before study procedures began. The research was approved by the institutional review boards of Cornell University, the University of Oklahoma, the Pennsylvania State University, and the Rwandan National Ethical Committee. The study was registered with clinicaltrials.gov under identifier NCT01594359.

#### Statistics.

All reaction times (RTs) were summarized for each subject at each time point as the median of the RTs for correct responses only. In addition, any RT that was <200 (anticipatory response) or >2000 ms (lapse of attention) on any trial was deleted. When needed to deal with heterogeneity of variance (33), accuracy variables were transformed using an arcsine square-root transformation. Log_10_ transformation was applied to SF before running tests. Chi-square tests were used to test for group (control vs. biofortified) differences at baseline in the proportion of women who were *1*) anemic (hemoglobin <120 g/L); *2*) iron deficient (≥1 of any of the following: SF <15 μg/L, TfR >8.3 mg/L, BI <0 mg/kg); *3*) anemic and iron deficient (hemoglobin <120 g/L and SF <15 μg/L); *4*) not anemic and not iron deficient (hemoglobin ≥120 g/L and SF <15 μg/L); and *5*) inflamed (AGP >1.0 g/L or CRP >5.0 mg/L).

The per protocol analyses for both blood and cognitive outcomes were done using generalized linear models with the endline value as the dependent variable, group (control or biofortified) as the independent variable, and the baseline value as a covariate.

In secondary analyses, linear regression was used to determine whether a change in hematological status (hemoglobin, SF, TfR, and BI) predicted a change in cognitive performance. These analyses involved fitting a null model (intercept only), a full model (fixed set of predictors), and a model whose final set of parameters was selected using stepwise model selection. The “best” model in each analysis needed to be significantly superior to the null model (as assessed by a χ^2^ test), had to have parameter estimates that were significantly different from 0, and had to account for ≥10% of the variance. Only women who were not anemic at baseline were included in this regression analysis because they were who we expected to show the greatest improvement in biomarkers specific to iron. For all regressions, highly influential points (Cook’s distance >1) were examined on a case-by-case basis and removed if the point was judged to be implausible (e.g., RTs well outside the 95% CI for the data; <0.1% of points were removed). All data were analyzed using SAS version 9.4 (SAS Institute, Inc.), and statistical significance was set at α <0.05.

## Results

**Table 1** shows the baseline characteristics of the women, 44% of whom were anemic (hemoglobin <120 g/L; of these, all but one also had low SF), 92% had low SF (<15 μg/L), 40% had elevated TfR (>8.3 mg/L), and 65% had negative BI (<0.0 mg/kg). Inflammation (either CRP >5 mg/L or AGP >1 g/L) was present in 11% of the women; adjusting the iron status markers for inflammation through the use of the method described by Thurnham et al. (34) did not alter any of the findings. Therefore, the values reported are unadjusted. No differences were found between treatment groups for demographic or hematological characteristics at baseline (**Tables 1** and **2**). In addition, no differences existed between this subsample and the larger sample from which these women were drawn (22) in terms of baseline demographic and hematologic characteristics (data not shown).

**TABLE 1:** Baseline demographic characteristics and prevalence of anemia, iron deficiency, iron deficiency anemia, and inflammation in Rwandan female college students randomly assigned to consume conventional or biofortified beans^1^

	Control group (*n* = 78)	Biofortified group (*n* = 72)
Age, y	22.3 ± 1.8	22.3 ± 1.6
BMI, kg/m^2^	22.3 ± 2.6	22.6 ± 2.9
Anemia (hemoglobin <120 g/L)	32 (41)	33 (46)
Iron deficiency		
Ferritin <15 μg/L	71 (91)	67 (93)
Transferrin receptor >8.3 mg/L	34 (44)	24 (33)
Body iron <0 mg/kg^2^	51 (65)	47 (65)
Iron deficiency anemia (hemoglobin <120 g/L and ferritin <15 μg/L)	32 (41)	32 (44)
Iron deficiency without anemia (hemoglobin ≥120 g/L and ferritin <15 μg/L)	39 (50)	35 (49)
Any inflammation^3^ (AGP >1.0 g/L or CRP >5.0 mg/L)	8 (11)	8 (12)

1 Values are means ± SDs or *n* (%). No significant group differences were found for any of the characteristics presented here. Note that only women with baseline ferritin <20 μg/L were included in the analyses, so the percentages in the table do not reflect the expected population prevalence. AGP, α-1-acid glycoprotein; CRP, C-reactive protein.

2 Calculated using Cook’s method (26).

3 Number of women with AGP and CRP data were 76 and 69, respectively.

**TABLE 2:** Summary of blood and cognitive outcomes at baseline and endline in Rwandan women randomly assigned to consume conventional or biofortified beans for 18 wk^1^

	Control group			Biofortified group		
Variables by outcome	Baseline	Endline	Difference	Baseline	Endline	Difference
Blood values						
Hemoglobin, g/L	121 (1)	120 (1)	0 (1)	119 (2)	122 (2)	4 (1)
Ferritin, μg/L	9.3 (0.4)	11.6 (0.6)	2.4 (0.5)	8.8 (0.4)	14.1 (0.9)	5.3 (0.8)
Transferrin receptor, mg/L	8.4 (0.5)	8.4 (0.4)	−0.1 (0.3)	8.3 (0.5)	8.4 (0.5)	0.0 (0.3)
Body iron,^2^ mg/kg	−1.1 (0.3)	−0.4 (0.3)	0.8 (0.2)	−1.2 (0.3)	0.2 (0.3)	1.5 (0.2)
SRT task RT, ms	280 (6)	273 (4)	−8 (7)	282 (7)	273 (5)	−6 (7)
GNG task RT, ms	373 (6)	347 (6)	−28 (6)	380 (8)	341 (5)	−38 (7)
ANT RT, ms						
0 Cues	617 (12)	572 (7)	−47 (11)	626 (13)	571 (9)	−54 (11)
2 Cues	579 (11)	447 (8)	−135 (11)	601 (14)	448 (10)	−154 (11)
Alerting	38 (5)	126 (5)	88 (6)	25 (7)	123 (6)	99 (9)
Center cue	601 (11)	545 (8)	−58 (10)	608 (14)	552 (9)	−58 (13)
Spatial cues	555 (10)	385 (7)	−173 (9)	562 (13)	369 (9)	−193 (11)
Orienting	46 (5)	161 (5)	115 (7)	46 (6)	183 (6)	135 (9)
Consistent flankers	600 (10)	545 (7)	−56 (9)	617 (15)	544 (9)	−74 (12)
Inconsistent flankers	770 (10)	646 (7)	−127 (10)	777 (16)	641 (9)	−139 (13)
Conflict	170 (7)	99 (6)	−71 (9)	160 (6)	97 (6)	−65 (7)
CRT						
Sensitivity (d′), SD	2.6 (0.1)	2.9 (0.1)	0.3 (0.1)	2.6 (0.1)	3.3 (0.1)	0.7 (0.1)
PCC, % change	63 (4)	106 (5)	44 (6)	59 (5)	133 (7)	74 (8)
RT, ms						
New items	829 (22)	822 (16)	−11 (20)	802 (21)	731 (16)	−75 (21)
Old items	787 (16)	805 (12)	14 (15)	790 (22)	712 (14)	−80 (22)
Bias (c), SD	−0.12 (0.04)	0.06 (0.03)	0.18 (0.05)	−0.13 (0.04)	−0.18 (0.04)	−0.05 (0.05)
SMS task RT						
Intercept, ms						
New	857 (25)	822 (14)	−40 (26)	856 (29)	730 (14)	−120 (30)
Old	852 (27)	818 (17)	−42 (26)	837 (30)	719 (18)	−105 (25)
Slope, ms/item						
New	50 (4)	54 (2)	4 (4)	47 (4)	34 (2)	−14 (4)
Old	50 (4)	52 (3)	3 (5)	47 (4)	30 (3)	−20 (5)

1 Values are raw means (SEs). ANT, attentional network task; CRT, cued recognition task; GNG, go/no-go; PCC, percentage change in capacity; RT, reaction time; SMS, Sternberg memory search; SRT, simple reaction time.

2 Calculated using Cook’s equation (26).

The mean hemoglobin concentration of the women in the 2 groups did not differ at baseline or endline, but women in the biofortified group experienced a greater increase in hemoglobin over time than did than women in the control group, who experienced a slight nonsignificant decrease over time (**Tables 2** and **3**). Although SF concentrations increased significantly in both groups from baseline to endline, the change in SF concentration was higher in women who consumed biofortified beans than in women who consumed conventional beans. The women who consumed biofortified beans also experienced an increase in BI over time such that the change in BI was significantly higher in these women than in those who consumed conventional beans.

**TABLE 3:** Per protocol analyses for blood and cognitive outcomes in Rwandan female college students randomly assigned to conventional or iron-biofortified beans for 18 wk^1^

	Baseline effect			Group effect		
Variables by outcome	*F*	MSE	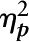	*F*	MSE	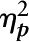
Blood						
Hemoglobin	578.3***	215	0.80	12.7***	5	0.08
Ferritin	119.1***	16	0.45	8.5**	1	0.06
Transferrin receptor	237.9***	830	0.63	0.1	0	0.00
Body iron^2^	278.4***	650	0.66	7.5**	18	0.05
SRT task RT	17.9***	21,812	0.11	0.0	5	0.00
GNG task RT	64.0***	100,546	0.31	1.4	2227	0.01
ANT RT						
0 Cues	79.8***	229,369	0.36	0.0	61	0.00
2 Cues	53.3***	212,450	0.28	0.6	2327	0.00
Alerting	5.5*	11,944	0.04	0.0	37	0.00
Center cue	78.6***	246,784	0.36	0.2	603	0.00
Spatial cues	64.3***	194,872	0.31	3.6^+^	10,828	0.02
Orienting	0.1	220	0.00	9.9**	19,353	0.07
Consistent flankers	92.1***	278,638	0.40	0.1	347	0.00
Inconsistent flankers	105.8***	297,102	0.43	0.3	703	0.00
Conflict	12.0***	27,639	0.08	0.0	59	0.00
CRT						
Sensitivity (d′)	2.6	1	0.02	15.1***	6	0.09
PCC	3.8^+^	9599	0.03	10.3**	26,278	0.07
RT						
New items	32.8***	487,279	0.18	16.6***	247,301	0.11
Old items	28.0***	294,491	0.17	27.4***	288,113	0.16
Bias (c)	1.1	0	0.01	24.4***	2	0.14
SMS task RT						
Intercept						
New	5.2*	72,774	0.04	19.7***	275,685	0.12
Old	27.5***	502,669	0.16	15.6***	284,449	0.09
Slope						
New	3.6^+^	1092	0.03	42.6***	12,779	0.23
Old	1.3	966	0.01	20.5***	15,594	0.13

1 Assessed using a generalized linear model regressing endline on group, controlling for baseline. ^+^*P* < 0.1; *P < 0.05; **P < 0.01; ***P < 0.001. ANT, attentional network task; CRT, cued recognition task; GNG, go/no-go; MSE, mean SE; PCC, percentage change in capacity; RT, reaction time; SMS, Sternberg memory search; SRT, simple reaction time; 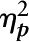, proportion of variance accounted for.

2 Calculated using Cook’s equation (26).

### Per protocol analyses.

Accuracy in the SRT task, GNG task, and ANT was uniformly high (women responded correctly on 99%, 98%, and 98% of trials for these 3 tasks, respectively), and preliminary analyses indicated no significant effects of the intervention on response accuracy. Therefore, we focus only on the RT variables for these tasks. Table 3 summarizes the results of the per protocol analysis for each dependent variable from each of the 5 cognitive tasks; means for each of the tasks are presented in Table 3. No significant group (treatment) effects were observed for the SRT or GNG task outcomes.

**FIGURE 3:**
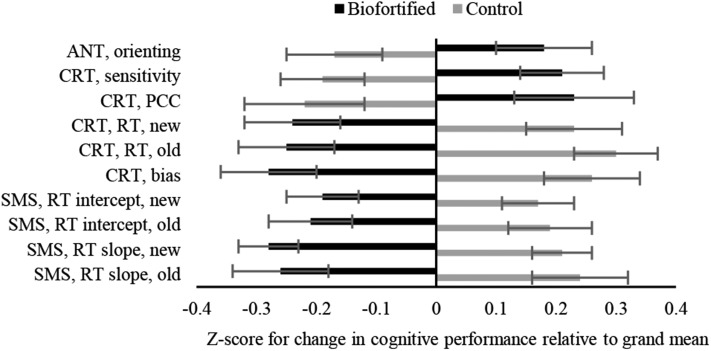
Change in behavioral performance of Rwandan female college students after consumption of conventional (control) or iron-biofortified beans for 18 wk. Data are shown as *z* scores with least square means and SEs. Positive or negative change indicates change more than or less than, respectively, the grand mean of change for the biofortified and control groups combined (does not indicate improvement or decrement, which is shown in Table 3). Only outcomes for which significant treatment effects were observed in per protocol analyses (Table 4) are shown. After bean consumption, the biofortified group showed more improvement than the control group in the control of spatial attention (ANT, orienting); sensitivity to the old or new status of a test item (CRT, sensitivity); capacity of memory retrieval (CRT, PCC); speed of memory retrieval and search (CRT, RT; and SMS task, RT intercept for both new and old items); liberal shift of response bias (higher probably of giving an “old” response to both types of stimuli; CRT, bias); efficient memory search (SMS task, RT slope). ANT, attentional network task; CRT, cued recognition task; PCC, percentage change in capacity; RT, reaction time; SMS, Sternberg memory search.

Only one variable from the ANT task (RT, orienting) showed a group effect with greater improvement in the biofortified group than in the control group. A significant main effect for group was seen for all outcomes from the memory-related CRT and SMS task. For the CRT, at the highest workload (4 cues), RT was reduced and bias became more liberal (more “old” responses given to both old and new stimuli) from baseline to endline among women who consumed biofortified beans, whereas RTs increased and bias became more conservative (fewer “old” responses given to both old and new stimuli) for women who consumed conventional beans. In terms of the ability to adapt to increasing workload, as indexed by the percentage change in capacity with increasing cues on the CRT, women in the group consuming iron-biofortified beans benefitted more from the increased workload over time than did those in the control group (Tables 2 and 3, **Figure 3**). For the SMS task outcome variables, the intercepts decreased more and the slopes became shallower (flatter) among women who consumed the biofortified beans compared with those who consumed the conventional beans (Tables 2 and 3, Figure 3), indicating that the speed and the efficiency of memory were improved.

### Secondary analyses to test plausibility.

**Table 4** summarizes results of the linear regression of change in hematological status on change in cognitive performance. Scatter plots with regression lines for hematological variables predicting ≥10% of the variability in cognitive performance change are shown in **Supplemental Figures 1–3**. Change in SF predicted change in performance for 9 variables across the ANT, CRT, and SMS task. Of these, 7 variables on 3 tasks (RT for the 0 cue, spatial cue, and inconsistent flankers trials on the ANT; RT for new and old items on the CRT; and RT intercept for new and old items on the SMS task) met our criteria for selecting a model. On all of these variables, RT decreased (women completed the tasks faster) as SF increased (as iron status improved). In the regression analyses, change in TfR was not related to change in cognitive performance. Change in the calculated BI concentrations was related to change in the same cognitive variables listed above for SF, suggesting that SF drives these relations (most likely a result of BI being driven by SF because we included only women with ID, not anemic women, in this analysis). The linear regression analyses involving the full model (including hemoglobin, SF, and TfR, or hemoglobin and BI as predictors) did not reach our criteria for the “best” model for any of the cognitive outcomes. After stepwise model selection, the following models met our criteria and were deemed to be the best models: models including change in log SF were best for predicting change in RTs on the ANT (0 and spatial cues), on the CRT (old items), and on the SMS task (intercept new and old); models including change in BI were best for predicting change in RTs for ANT (inconsistent flankers) and for CRT (new items); models including change in hemoglobin were best for predicting change in RT for the SMS task (slope). No acceptable model was found for the other cognitive outcome variables. Table 4 shows estimates for the individual predictors to allow comparison.

**TABLE 4:** Regression of change in cognitive performance on change in blood measures among nonanemic Rwandan female college students in the efficacy trial, irrespective of treatment assignment^1^

	Ferritin^2^			Transferrin receptor			Body iron		
Variables by task	Intercept	β	*R*^2^	Intercept	β	*R*^2^	Intercept	β	*R*^2^
SRT task RT	−11.3	7.1	0.00	−8.7	−2.1	0.01	−11.1	2.1	0.00
GNG task RT	−27.5	−19.5	0.03	−33.4	−2.2	0.01	−30.4	−3.4	0.01
ANT RTs									
0 Cues	−24.4	−69.7**	0.14^#^	−46.3	−2.4	0.00	−32.4	−14.0*	0.09
2 Cues	−124.7	−50.0	0.06	−140.9	3.2	0.00	−127.6	−12.8	0.06
Alerting	93.9	0.9	0.00	95.5	−6.4	0.03	90.0	4.1	0.01
Center cue	−40.3	−52.2	0.05	−57.5	0.2	0.00	−44.4	−12.6	0.04
Spatial cues	−157.2	−91.0***	0.17^#^	−187.8	3.3	0.00	−163.9	−22.3***	0.15
Orienting	116.7	38.5	0.05	129.9	−3.1	0.01	119.2	9.7	0.05
Consistent flankers	−42.2	−50.5*	0.08	−58.0	0.7	0.00	−46.4	−11.8^+^	0.07
Inconsistent flankers	−98.4	−84.4***	0.17	−126.9	9.9	0.05	−98.2	−26.4***	0.26^#^
Conflict	−63.9	−19.3	0.01	−71.4	5.7	0.02	−62.2	−7.8	0.03
CRT									
Sensitivity (d’)	0.3	0.7*	0.08	0.5	0.0	0.00	0.3	0.16*	0.07
PCC	44.7	23.9	0.02	52.9	−2.5	0.00	44.9	7.4	0.04
RTs									
New items	17.1	−171.1**	0.13	−43.5	21.4	0.04	11.0	−48.1***	0.16^#^
Old items	40.1	−168.8***	0.23^#^	−16.0	3.8	0.00	28.0	−42.2***	0.22
Bias (c)	0.1	−0.1	0.01	0.1	0.0	0.00	0.1	0.0	0.00
SMS task RTs									
Intercept									
New	10.6	−223.8***	0.16^#^	−61.5	−6.0	0.00	−13.3	−47.4**	0.11
Old	3.9	−221.5***	0.17^#^	−67.9	−4.3	0.00	−19.5	−47.3**	0.12
Slope									
New	4.0	−20.1	0.04	−3.0	1.8	0.01	3.3	−5.6	0.05
Old^3^	2.5	−18.8	0.04	−3.7	0.2	0.00	1.0	−4.5	0.03

1 Nonanemic was defined as hemoglobin ≥120 g/L (*n* = 84). ^+^*P* < 0.1; *P < 0.05; **P < 0.01; ***P < 0.001 compared with an intercept-only model, χ^2^ test; individual predictors were also tested. ^#^These were the “best” models, which predicted ≥10% of the variance in the outcome and were significant (*P* ≤ 0.05) compared with the intercept-only model based on a χ**^2^** test. ANT, attentional network task; CRT, cued recognition task; GNG, go/no-go; PCC, percentage change in capacity; RT, reaction time; SMS, Sternberg memory search; SRT, simple reaction time.

2 Ferritin was log-transformed.

3 The “best” model for SMS RT, slope, old items used change in hemoglobin as a predictor.

## Discussion

This study is, to our knowledge, the first to examine relations between changes in iron status indicators and multiple cognitive outcomes in WRA as a result of consuming meals including an iron-biofortified crop. The design of the study, a randomized controlled trial, provides strong evidence for a causal linkage between consumption of iron-biofortified beans and the measured cognitive improvements. As in the larger parent trial (22), we found that consumption of iron-biofortified beans significantly improved the iron status of Rwandan WRA. Furthermore, we found significant treatment effects for multiple memory outcomes and one attention outcome when comparing those consuming iron-biofortified beans with those consuming conventional beans. Examination of the amount of variance accounted for indicated that the intervention had a relatively larger impact on the memory tasks than on the other tasks, specifically on the efficiency of search and the speed of retrieval. To support these findings, our data demonstrated that improvements in ferritin were related to improvements in performance on tasks of attention and memory. In terms of academic achievement, the memory tasks assess aspects of memory retrieval that are pertinent to tasks such as recognizing and identifying anatomical parts and their functions, and patterns of symptoms indicative of medical conditions (note the relevance to the study participants, who were all medicine majors).

We have corroborated our and others’ previous findings and expanded them by showing that consumption of iron-biofortified beans daily for 18 wk has the potential to benefit cognition, even at low doses of iron. Two observational studies reported relations between iron biomarkers and planning time for an executive functioning task (9, 10), as well as RT on an attention task and inhibitory control task (10), in women attending university. In addition, previous work with WRA showed that cognitive outcomes are responsive to iron treatment, but nearly all of these studies provided treatment in the form of iron supplementation, which provides higher doses than what the women received in the present study (13–16, 35, 36). To our knowledge, only one study to date has tested the efficacy of a naturally iron-rich food (beef) in improving cognitive performance (37). Although those authors reported no difference in cognitive scores for the group consuming beef at lunch compared with those not consuming beef at lunch (iron concentrations improved in women in both groups), they found a relation between BI concentrations and spatial working memory and planning speed. Furthermore, women who experienced an improvement in ferritin concentrations had greater improvements in planning speed, spatial working memory strategy, and attention than did women who did not experience an improvement in ferritin concentrations. Our findings that RT (speed) on memory tasks improves with consumption of iron-biofortified beans and that increases in SF concentrations are related to faster RTs on attention and memory tasks support the findings of Blanton (37).

No associations were observed between treatment and changes in performance on the SRT or GNG tasks, or between changes in iron biomarkers and performance on these 2 tasks. In addition, although the ANT measure specific to spatial attention (orienting) was significantly related to SF, none of the other ANT measures showed significant relations with any of the blood markers for iron. One possible reason for the lack of significant relations may be that the tasks simply did not sufficiently tax these specific subjects to the degree that a relation might be observed. Specifically, performance on the SRT task, GNG task, and ANT was extremely good, in that accuracies were near the ceiling and RTs were near the floor. This may simply be because the participants are among the most educated in their country and are at an age when RTs are near their life-span minimum (38, 39). This suggests that the tasks may need to be more challenging in order to detect differences in perceptual and attentional function in similar populations. Neural correlates of these particular tasks may be unrelated to iron status or may be unaffected until the ID progresses to a more severe stage than the iron status of subjects in our current study sample, and we are currently exploring this possibility in a companion data set.

Consistent with the literature from animal studies, the observed cognitive deficits are likely related to striatal, hippocampal, and prefrontal cortex functioning, and changes in neurotransmitter signaling pathways could underlie the performance differences. Dopaminergic and serotonergic signaling within the mesolimbic, mesocortical, and nigrostriatal pathways are known to be iron dependent (40–43). Unfortunately, the present data set cannot speak in any informed way to this possibility. However, it would be possible to test this possibility by taking advantage of behavioral measures that deal directly with the differential involvement of these systems (44), particularly if done with concurrent measures of brain dynamics (such as electroencephalography).

The strengths of our study include the randomized controlled design, trial duration sufficient to detect changes in iron biomarkers, measurement of cognitive and iron statuses at multiple time points for each woman, measurement of multiple iron biomarkers, assessment of inflammation, a sufficient sample size, and the use of a battery of 5 cognitive tasks to assess several cognitive dimensions. Another strength is the effectiveness of the randomization, evidenced by no differences between any groups on any baseline measures. The primary limitation is the homogeneous nature of the sample; we recruited only WRA who were attending the University of Rwanda, and thus the generalizability of our findings may be limited beyond the proof of concept that was tested in this study.

In conclusion, these findings, considered in the context of past findings, indicate that cognitive performance is sensitive to iron status in WRA. Furthermore, we show here—to our knowledge for the first time—that treatment with a biofortified staple food crop can improve cognitive performance. These findings add to the growing literature indicating that detrimental consequences of ID occur in the mature brain and are not limited to the developing brain. In young adults, it seems that the time from adolescence to early adulthood is a potentially sensitive period with respect to the maturation and connectivity of the frontal cortex (45). In addition, as noted above, the differential involvement of the major dopaminergic pathways suggests the possibility that differences in the types of learning that may be required in particular domains, such as science, technology, engineering, and mathematics disciplines, may be differentially sensitive to the effects of ID (44). Given that ID disproportionately affects WRA (the age range of most of the women attending universities) and may differentially pose a challenge to women in the science, technology, engineering, and mathematics disciplines, additional research that aims to understand the ramifications of ID, particularly mild ID in the absence of anemia, in young adult women is needed.
